# Poisson’s Ratio of the f.c.c. Hard Sphere Crystals with Periodically Stacked (001)-Nanolayers of Hard Spheres of Another Diameter

**DOI:** 10.3390/ma12050700

**Published:** 2019-02-27

**Authors:** Jakub W. Narojczyk, Krzysztof W. Wojciechowski

**Affiliations:** Institute of Molecular Physics, Polish Academy of Sciences, M. Smoluchowskiego 17, 60-179 Poznań, Poland; kww@ifmpan.poznan.pl

**Keywords:** auxetics, negative Poisson’s ratio, nanolayers, hard sphere inclusions, Monte Carlo simulations

## Abstract

The results of studies on the influence of periodically stacked nanolayer inclusions, introduced into the face-centered cubic (f.c.c.) hard sphere crystal, on Poisson’s ratio of the obtained nanocomposite system are presented. The monolayers are orthogonal to the [001]-direction. They are formed by hard spheres with diameter different from the spheres forming the matrix of the system. The Monte Carlo computer simulations show that in such a case the symmetry of the system changes from the cubic to tetragonal one. When the diameter of the inclusion spheres increases at certain range, a decrease of the negative Poisson’s ratio in the $\left[101\right]\left[\bar{1}01\right]$-directions is observed, i.e., the system enhances its partial auxeticity. The dependence of the maximal, average, and negative parts of the minimal Poisson’s ratio on the direction of the applied load are shown in a form of surfaces in spherical coordinates, plotted for selected values of nanolayer particle diameters. The most negative value of the Poisson’s ratio found among all studied systems was −0.11 (at pressure $p^{*}=100$, which is about ten times higher than the melting pressure) what is almost twice more negative than in the f.c.c. crystal of identical hard spheres. The observed effect weakens along with the decrease of pressure and becomes hardly noticeable near melting. This study indicates that modifying only the size of the inclusion particles one can change Poisson’s ratio of nanocomposites at high pressures.

## 1. Introduction

This work presents results of research regarding elastic properties of a hard sphere system with a periodic stack of monolayer inclusions oriented perpendicularly to [001]-direction. The selection of the hard potential, infinite when any pair of particles overlap and zero otherwise, is not incidental. The model of hard spheres is the simplest example of the class of hard body models which are governed by the entropy [1]. It serves as a fundamental reference model for melting and theory of liquids [2]. Moreover, models with purely geometrical, hard interactions between particles are athermal. As a result, their phase diagrams are simplified as the pressure, *p*, and the temperature, *T*, occur only as the p/T ratio. In the theory of elasticity, the hard potential acts as the limiting case for anharmonic interactions. For these reasons, various properties and behaviors of hard spheres were the subject of intense studies [3,4,5,6,7,8,9,10,11,12,13,14,15]. As an example, one might refer to the study of elastic properties of the face-centered cubic (f.c.c.) crystal of hard spheres, where it has been shown that by increasing concentration of vacancies one can modify its elastic properties, in particular increase Poisson’s ratio (PR) [16]. The PR is a negative ratio of the relative changes of lateral to longitudinal dimensions of a body subjected to an infinitesimal change of uniaxial stress applied in the longitudinal direction, and is one of the parameters that characterize how materials deform when subjected to an external stress [17]. The value of Poisson’s ratio can vary in the range between −1 and 1/2 for isotropic three-dimensional systems. However, for most of the materials that we know from our surrounding, its value falls in the range between 0 (e.g., cork) and ∼0.5 (e.g., rubber) [17].

For anisotropic systems Poisson’s ratio changes with the direction of measurement and may take any value between minus and plus infinity [18]. A relatively recently discovered group of materials for which Poisson’s ratio takes negative values [19], the so-called *auxetics* [20], has become the subject of intense studies, both theoretical [21,22,23,24,25,26,27,28,29,30,31,32,33,34,35,36,37,38,39,40,41,42,43,44,45,46,47,48,49,50,51,52,53,54,55,56,57,58] as well as experimental [59,60,61,62,63,64,65,66,67,68,69,70,71,72,73,74,75,76,77]. This is due to their extraordinary, counter intuitive, elastic behavior and the potential applications of these materials [78,79,80,81,82,83,84].

One of the directions of research on auxetic materials is the analysis of simple atomic models with structural-level modifications and the influence of the latter on the material’s elastic properties. Not long ago, such nanoscale models were out of reach in the sense of preparation and, hence, also for practical applications. However, recent development of methods for nanostructure fabrication allows one to manufacture quasiplanar metamaterials at nanoscale, e.g., by atomic layer deposition techniques [85]. This allows one for fabrication of nanolayer [85] or nanowire [86,87] metamaterials. Therefore, it is meaningful to search for such modifications at the nanoscale that lead to the decrease of Poisson’s ratio.

It has been recently shown that a single layer of hard spheres introduced into the f.c.c. crystal of Yukawa spheres (which in its pristine form exhibits weak auxetic properties [88]) significantly decreases the Poisson’s ratio in the [001]-direction when the external stress is applied in the [100]-direction [89]. In this work we consider only hard interactions to answer the more fundamental question: is there any, and if so, what is the influence of the inclusion particles’ size (in relation to the size of the remaining particles forming the matrix of the system) on Poisson’s ratio of the model system? The results of the study are compared with the results obtained for the f.c.c. crystal composed of identical hard spheres.

The work is organized as follows: the details of the studied model are described in the next section. In Section 3 the simulation method and the calculations of the elastic properties in the NpT ensemble, as well as the details concerning the parameters of simulations are presented. Section 4 contains the discussion of the obtained results, whereas in the last part (Section 5) the summary and conclusion are included.

## 2. The Model Studied

**Figure 1:**
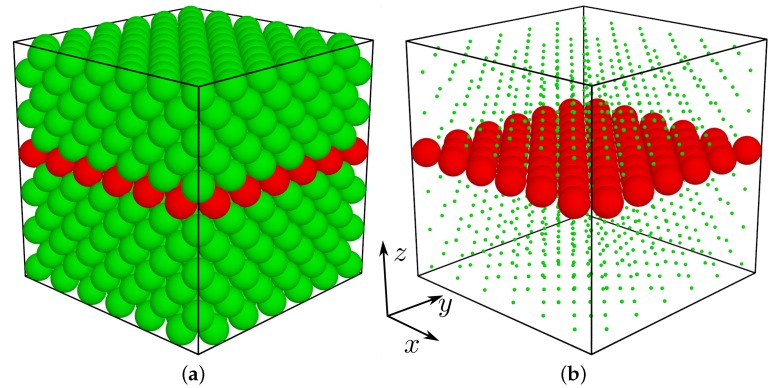
The initial (f.c.c.) unit supercell with the inclusion particles drawn in red (**a**). The positions of the matrix atoms have been scaled down on subfigure (**b**) to reveal the structure of the inclusion.

In this work the model of *N* spheres (initially) forming the f.c.c. lattice is considered. In the crystal without inclusions all the spheres have the same diameter $\sigma_{i}=\sigma$ which constitutes the unit of length where *i* is the index of the *i*-th sphere. Each sphere interacts with other spheres through the well-known hard sphere (HS) potential of the form:

(1) $$\beta u_{ij}=\left\{\begin{array}{cc} \infty , & r_{ij}<\sigma_{ij},\\ 0, & r_{ij}\geq \sigma_{ij}, \end{array}\right.$$

where $r_{ij}$ is the distance between the centers of spheres *i* and *j*, $\sigma_{ij}=\left(\sigma_{i}+\sigma_{j}\right)/2$, $\beta =1/(k_{B}T)$, $k_{B}$ is the Boltzmann constant, and *T* is the temperature. Into such a system we introduce an inclusion by selecting (at close packing limit) an arbitrary crystalline plane orthogonal to [001]-direction (see Figure 1). Spheres in the plane constitute the inclusion further referred to as the nanolayer. The number of spheres in that plane ($N_{L}$) depends only on the size of the studied system in the directions *x* and *y*, and is equal to $2N_{x}N_{y}$ (the latter being the numbers of the f.c.c. cells in the respective directions). Such a system will be thought of as a unit supercell. Due to applied periodic boundary conditions, effectively we obtain a system with a periodic stacking of (infinite in the *x* and *y* direction) supercells along the *z*-axis. The ratio of the number of spheres forming the single nanolayer to the number of all the particles in the system will be referred to as the concentration $c=(N_{L}/N)\times 100\%$ [90]. Both the inclusion particles and the matrix particles interact with the HS potential (1). The nanolayer particles differ from the matrix particles only with respect to their diameters $\sigma^{\prime }\neq \sigma$. The diameters of all the inclusion particles are equal to each other. Thus, one obtains a binary composite, similar to the model studied in [89]. The influence of the changes of the inclusion’s particle diameter ($\sigma^{\prime }$) on the elastic properties of such a composite is investigated.

## 3. Theory and Method

### 3.1. Elastic Properties

The Monte Carlo (MC) computer simulations in the NpT ensemble (with constant number of particles, at constant pressure and temperature) and the Parrinello–Rahman [91,92,93] method with the variable shape of the periodic box have been used to determine the elastic compliance tensor elements $S_{\alpha \beta \gamma \delta }$ of the model described in the previous section. This method allows to calculate the latter directly from the shape fluctuations of the periodic box, which is described by a *symmetric* matrix h, formed by vectors defining the edges of the parallelepiped (periodic box) containing the studied system. The strain tensor ε can be determined from the following formula [92,93]:

(2) $$\varepsilon =\frac{1}{2}\left(h_{0}^{-1}.h.h.h_{0}^{-1}-I\right)\;,$$

where I is the unit matrix of the dimension three, $h_{0}$ is the reference box matrix (equilibrium matrix h at pressure $p^{*}$, $h_{0}\equiv \langle h\rangle$). The elastic compliance tensor can be expressed in relation to the strain tensor [93]:

(3) $$S_{\alpha \beta \gamma \delta }=\beta V_{p}\langle \Delta \varepsilon_{\alpha \beta }\Delta \varepsilon_{\gamma \delta }\rangle ,$$

where $V_{p}=\left|\det\left(h_{0}\right)\right|$ is the volume of the system at pressure $p^{*}$, $\Delta \varepsilon_{\alpha \beta }=\varepsilon_{\alpha \beta }-\langle \varepsilon_{\alpha \beta }\rangle$, $\langle \varepsilon_{\alpha \beta }\rangle$ is the average in the NpT ensemble, and α,β,γ,δ = *x*, *y*, or *z*.

**Figure 2:**
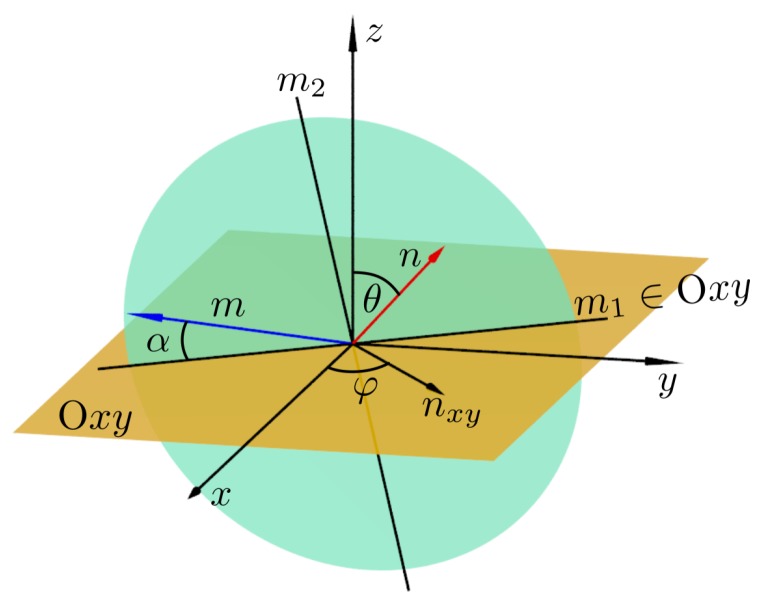
The direction of the applied stress $\vec{n}$ as a function of polar and azimuthal angles θ,φ, and the direction in which the Poisson’s ratio is measured $\vec{m}$ as a function of α-angle lying in the plane (presented in the form of a green disc) spanned by the vectors $m_{1}=\vec{n}\times \vec{z}$ and $m_{2}=\vec{n}\times m_{1}$. All vectors are unit vectors.

The knowledge of all the 21 independent elastic compliance tensor components allows one for the complete description of elastic properties and for calculation of Poisson’s ratio (ν) in arbitrary direction for crystals of arbitrary symmetry. In the general case, Poisson’s ratio depends on the pair of directions $\vec{n}$ and $\vec{m}$. The former is the one in which the external stress is applied. The latter is the direction in which the Poisson’s ratio is measured. One should note that the $\vec{m}$-direction is located on the plane perpendicular to the $\vec{n}$-direction ($\vec{n}\cdot \vec{m}=0$). The examples of these directions have been illustrated in Figure 2. The following formula [94] has been used to calculate the Poisson’s ratio for selected pair of directions $\vec{n}$ and $\vec{m}$:

(4) $$\nu_{nm}=-\frac{m_{\alpha }m_{\beta }S_{\alpha \beta \gamma \delta }n_{\gamma }n_{\delta }}{n_{\zeta }n_{\eta }S_{\zeta \eta \kappa \lambda }n_{\kappa }n_{\lambda }},$$

where n,m are indexes corresponding to $\vec{n}$ and $\vec{m}$ vectors respectively, and $n_{\alpha },m_{\beta }$ are their respective direction cosines. Further in the work we replace the $S_{\alpha \beta \gamma \delta }$ tensor with a symmetric square matrix S of the dimension six, using Voigt representation [95]. Thus, i,j indexes for $S_{ij}$ elements take the values i,j=1,…,6. By convention, Einstein summation is used on Greek indexes. It should be also added that in this work we determine Poisson’s ratio only for infinitesimally small strains. The case of a large deformations will be the subject of the future studies with the use of the method described in [24].

### 3.2. Details of Computer Simulations

The size of the simulated samples has been equal to 6×6×6 f.c.c. unit cells, i.e., N=864 spheres, $N_{L}=72$ of which formed the inclusion. Thus, the concentration of the inclusion particles was c=8.33%. It has been verified that doubling or quadrupling the sample in the z-direction, what can be thought of as simulation of two- or four-unit supercells, influences the results at the order of the experimental error. The simulations have been mainly performed at pressure $p^{*}=\beta p\sigma^{3}=100$. This value was selected to enable comparison between the new results with the ones obtained from earlier study on another model system [89]. Although the selected pressure might seem high, it is worth mentioning that it is only by an order of magnitude higher than the pressure at which the system of HSs undergoes melting. Additionally, some results have been obtained for lower pressures. The elastic properties were studied for various diameters of particles inside the nanolayer ranging from $\sigma^{\prime }/\sigma =0.95$ to $\sigma^{\prime }/\sigma =1.054$. The values of diameters were small enough to avoid melting of the system. The stability of the studied systems was verified by simulations of samples with doubled sizes respectively in each direction (6×6×12, 12×6×6 and 12×12×6) for selected diameters $\sigma^{\prime }/\sigma =0.95,\;1.025,\;1.05$. The results of these simulations agree with the results for 6×6×6 systems within the range of the experimental error. The results were compared with elastic properties of the cubic system of HSs ($\sigma^{\prime }/\sigma =1$) [16]. The diameters of inclusion spheres were constant during individual simulations. For each value of the ratio $\sigma^{\prime }/\sigma$ ten independent simulations were conducted, each of the length equal to $10^{7}$ MC cycles, of which the first $10^{6}$ cycles were discarded prior to calculations of physical quantities. The verifying simulations of larger systems have been conducted independently 20 times for each $\sigma^{\prime }/\sigma$ and lasted $2\times 10^{7}$ MC cycles for 6×6×12, 12×6×6 and $5\times 10^{7}$ MC cycles for 12×12×6 systems.

## 4. Results and Discussion

**Figure 3:**
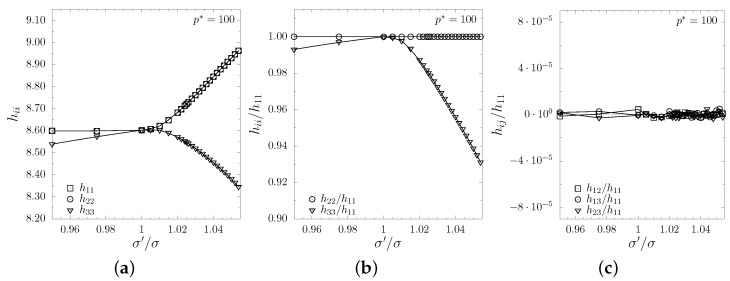
The (**a**,**b**) diagonal and (**c**) off-diagonal box matrix elements. In (**b**,**c**) plot the respective $h_{ii}$ and $h_{ij}$ elements have been divided by $h_{11}$ values, to show that the shape of the box corresponds to the tetragonal symmetry. The lines are drawn to guide the eye.

**Figure 4:**
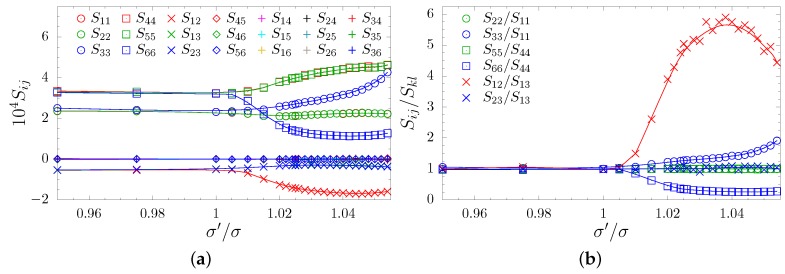
The elastic compliance matrix elements: (**a**) plotted as a function of the $\sigma^{\prime }/\sigma$ ratio and (**b**) the selected ratios of elastic compliance elements plotted to show that the respective relations imposed by the symmetry are fulfilled. The lines are drawn to guide the eye.

Since both the inclusion particles and the matrix particles interact with hard potential, the system in which $\sigma^{\prime }=\sigma$ is equivalent to HS crystal with respect to the box shape and the elastic properties. In Figure 3 one can observe (based on the averaged elements of the box matrix) the influence of the $\sigma^{\prime }/\sigma$ on the shape of the periodic box. The plots show that the box changes its shape from cubic to parallelepiped with square base (orthogonal to O*z* axis), described by the matrix:

(5) $$h_{0}\equiv \langle h\rangle =\left[\begin{array}{ccc} \langle h_{11}\rangle & 0 & 0\\ 0 & \langle h_{11}\rangle & 0\\ 0 & 0 & \langle h_{33}\rangle \end{array}\right].$$

where an increase of the inclusion particles’ diameters, $\sigma^{\prime }/\sigma >1$, forces an increase of the distances between the matrix particles in planes parallel to the inclusion planes and a compression of the layers in the direction orthogonal to the inclusion planes. This is manifested in the changes of the interparticle distances. When $\sigma^{\prime }/\sigma <1$, the above-mentioned compression of the matrix layers is not possible. Thus, only a small compression, resulting from the fact that one of the layers’ height has been decreased, is observed. To determine the symmetry of the system, all 21 elements of the elastic compliance matrix S as well as their selected ratios have been determined and presented as functions of $\sigma^{\prime }/\sigma$, see Figure 4a,b, respectively. One can observe that the relations typical for the cubic symmetry ($S_{11}=S_{22}=S_{33}$, $S_{44}=S_{55}=S_{66}$, and $S_{12}=S_{13}=S_{23}$) are no longer preserved when $\sigma^{\prime }/\sigma >1$. One can notice that $S_{33}$ increases, whereas the $S_{12}$ and $S_{66}$ decrease respectively in relation to $S_{11}$, $S_{13}$, and $S_{44}$ (see Figure 4b). The $S_{12}/S_{13}$ is greater than 1 because both elements are negative and $|S_{12}\left|>\right|S_{13}|$. Moreover, in Figure 4b one can see that the equalities of $S_{11}=S_{22}$, $S_{44}=S_{55}$ and $S_{13}=S_{23}$ are preserved, as well as the fact that $S_{ij}=0$ for: i=1,…,6, j=4,5,6, i≠j (Figure 4a). Thus, the S matrix takes the form typical to tetragonal symmetry [95]:

(6) $$S=\left[\begin{array}{cccccc} S_{11} & S_{12} & S_{13} & 0 & 0 & 0\\ \cdot & S_{11} & S_{13} & 0 & 0 & 0\\ \cdot & \cdot & S_{33} & 0 & 0 & 0\\ \cdot & \cdot & \cdot & S_{44} & 0 & 0\\ \cdot & \cdot & \cdot & \cdot & S_{44} & 0\\ \cdot & \cdot & \cdot & \cdot & \cdot & S_{66} \end{array}\right],$$

with six independent elements $S_{11}$, $S_{33}$, $S_{44}$, $S_{66}$, $S_{12}$, and $S_{13}$. Despite not observing any significant changes in the $S_{ij}$ elements for $\sigma^{\prime }/\sigma <1$, one must remember that in each case when $\sigma^{\prime }/\sigma \neq 1$ the symmetry of the system is not cubic because of the missing 4-fold symmetry axes in *x* and *y*-directions.

**Figure 5:**
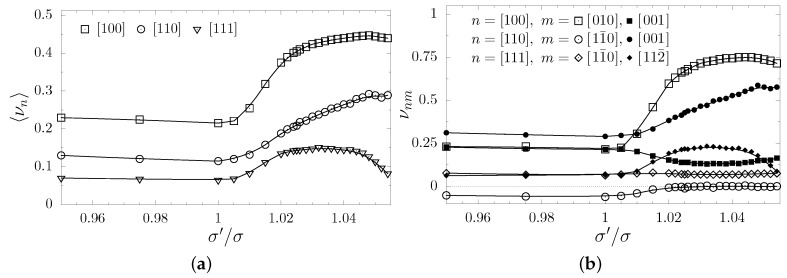
The Poisson’s ratio for systems with (001)-nanolayers plotted as a function of $\sigma^{\prime }/\sigma$ ratio for selected crystallographic directions (**a**) averaged over all $\vec{m}$-directions respectively and (**b**) measured in two mutually orthogonal $\vec{m}$-directions (marked pairwise with open or filled symbols). The lines are drawn to guide the eye.

It is interesting how the change of the symmetry from cubic to tetragonal one (422 symmetry class [95]), caused by the presence of the nanolayer, impacts the elastic properties of the system. In Figure 5a the Poisson’s ratios averaged over all $\vec{m}$-directions, for selected $\vec{n}$-directions: [100], [110] and [111] is presented. The first and the last of the listed $\vec{n}$-directions are the, so-called, high symmetry directions in the cubic system, i.e., the value of the Poisson’s ratio in such directions does not depend on the $\vec{m}$-direction (what can be seen in Figure 5b). One can observe there that $\langle \nu_{n}\rangle$ increases along with the increase of $\sigma^{\prime }/\sigma$ in all three directions. Thus, one might ask whether an increase of the inclusion particles’ sizes causes a general increase of Poisson’s ratio. To determine this, the values of the Poisson’s ratios have been plotted for pairs of (mutually orthogonal) $\vec{m}$-directions, for selected $\vec{n}$-directions. As it can be seen in the Figure 5b, the Poisson’s ratio typically increases for $\sigma^{\prime }/\sigma >1$. Likewise, in the $\left[110\right]\left[1\bar{1}0\right]$-directions, the Poisson’s ratio value becomes positive around $\sigma^{\prime }/\sigma =1.03$ (it is worth noting that PR is negative in the cubic system in this direction). Only a minor decrease of the Poisson’s ratio is observed in the [100][001]-directions.

**Figure 6:**
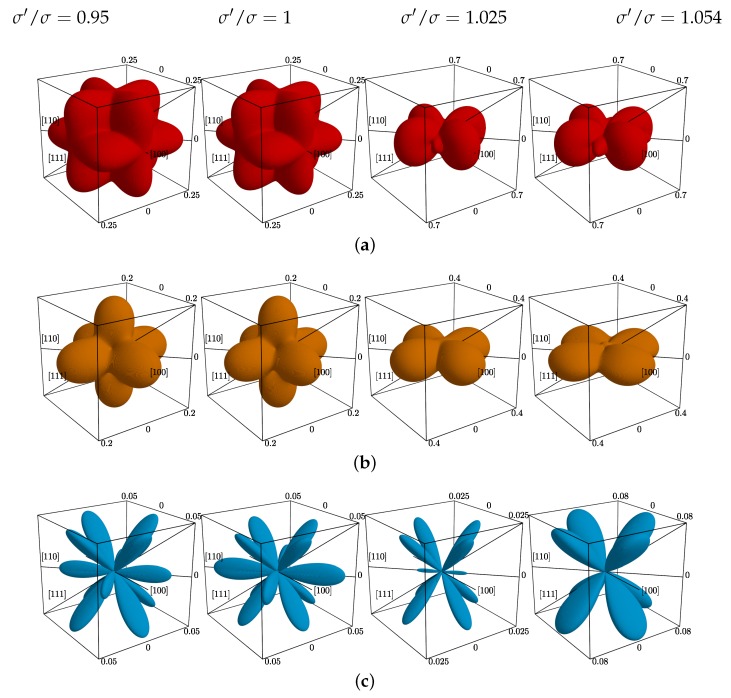
The array of plots of the Poisson’s ratio for selected values of $\sigma^{\prime }/\sigma$ ratio (in respective columns). The rows contain: (**a**) maximal PR, (**b**) average PR, and (**c**) negative part of the minimal Poisson’s ratio in $\vec{n}$-directions. For each plot, the infinite set of possible $\vec{n}$-direction has been uniformly sampled in $5\times 10^{5}$ distinct directions.

Based on the above discussion , at a first glimpse, one might expect that overall auxetic properties weaken with increasing $\sigma^{\prime }/\sigma$. To investigate this, the surfaces of the Poisson’s ratio in the spherical coordinate system have been plotted (ν values have been sampled in ~$5\times 10^{5}$ directions for each plot). Figure 6 presents: (a) the surfaces of maximal, (b) average, and (c) the negative part of the minimal Poisson’s ratio for selected values of $\sigma^{\prime }/\sigma$ (in respective columns). The $\vec{n}$-directions (in which the external stress is applied) are represented on each plot by polar and azimuthal angles θ and φ (see Figure 2), and the distance *l* between the point on the surface and the origin of the coordinate system is, respectively, equal to $\nu_{n}^{\max}$ for (a), $\langle \nu_{n}\rangle$ for (b), and the following for (c):

(7) $$l=\left\{\begin{array}{cc} |\nu_{n}^{\min}| & \mathrm{when}\;\nu_{n}^{\min}<0,\\ 0 & \mathrm{when}\;\nu_{n}^{\min}\geq 0. \end{array}\right.$$

**Figure 7:**
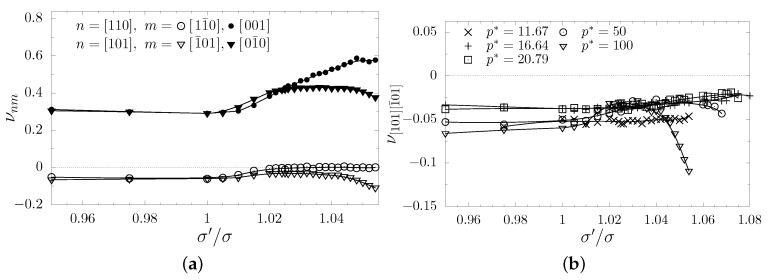
(**a**) The comparison of the Poisson’s ratio the [110]- and [101]-directions, which are equivalent in cubic systems ($\sigma^{\prime }/\sigma =1$), plotted as a function of $\sigma^{\prime }/\sigma$ ratio. (**b**) The dependence of Poisson’s ratio in the $\left[101\right]\left[\bar{1}01\right]$-direction on the external pressure conditions. The lines are drawn to guide the eye.

It can be seen that auxetic properties *do not* vanish in the tetragonal systems with nanolayers. However, one can notice that for $\sigma^{\prime }/\sigma =1.025$ they are significantly weaker than, e.g., in the cubic system. After a closer examination of Figure 6c one can see that the negative values of the Poisson’s ratio are of the same order of magnitude as in the pristine cubic system. The auxetic properties vanish in the directions laying in the xy-plane, but are enhanced in xz- and yz-planes. Thus, in Figure 7a the values of the Poisson’s ratio for the cases when the external stress was applied in [110] and [101]-directions have been compared (these directions are equivalent in the cubic system). It can be seen that the increase of $\sigma^{\prime }/\sigma$ value causes the increase of the Poisson’s ratio. However, above the $\sigma^{\prime }/\sigma =1.03$ Poisson’s ratio decreases in the $\left[101\right]\left[\bar{1}01\right]$-directions, reaching the value of −0.11 for $\sigma^{\prime }/\sigma =1.054$ at pressure $p^{*}=100$, which is almost two times lower than −0.059 obtained for the cubic system (the value $\sigma^{\prime }/\sigma =1.054$ was the highest diameter of the inclusion particles studied in this work for $p^{*}=100$). Additionally, Poisson’s ratio in the $\left[101\right]\left[\bar{1}01\right]$-directions has been computed for lower external pressures (even close to melting). The data have been presented in Figure 7b (the data for $p^{*}=100$ are the same as in Figure 7a). It can be noticed that at lower pressures one requires higher $\sigma^{\prime }/\sigma$ values to achieve a decrease of PR. In the case of $p^{*}=20.79,\;16.67$, or 11.67 [96] one does not observe any significant effect in contrast to what was seen in the case of $p^{*}=100$. It seems that the dimensions of the inclusion particles are too small. However, further increase of $\sigma^{\prime }/\sigma$ forces the system at these pressures to undergo phase transition and to melt.

This study indicates that the effects of the auxeticity enhancement for the considered geometry of the system is pronounced when the relative size of the inclusion and matrix particles exceeds the ratio of the average distance between the centers of particles’ positions under given pressure and at close packing.

The elastic properties determined above for the HS model system at $p^{*}=100$ have been compared with the results obtained for Yukawa model by Pigłowski et al. [89]. The discussion of elastic properties in that work concerned the f.c.c. crystal of particles interacting with hard-core repulsive Yukawa potential, in which one of the crystalline Yukawa layers, oriented orthogonally to the [010]-direction, has been replaced with a layer of HSs. The Yukawa particles interacted with inclusion particles only through their hard cores. The elastic properties of such a system have been studied as a function of the concentration parameter *c* (the ratio of hard particles to all the particles in the system). The authors influenced elastic properties by changing the number of Yukawa layers between consecutive inclusion layers (effectively changing the concentration). They showed that by applying the in-plane stress (in the direction of main axis parallel to the inclusion plane), one can observe a decrease of the out-of-plane (measured in the direction orthogonal to the inclusion plane) Poisson’s ratio accompanied by an increase of the in-plane Poisson’s ratio (measured in the direction parallel to the inclusion plane and orthogonal to the direction of applied stress). It has been shown that for the highest concentration, c=16.67%, the out-of-plane PR reaches −0.57 which is significantly lower than the value ≈+0.43 obtained for a pure Yukawa system (c=0%). In the HS system, in a current study, we selected an average c=8.33% which yield the minimum Poisson’s ratio ν≈−0.27 in Yukawa system. We wanted to check if by purely geometrical effects (changes of the particles’ sizes) an analogous change in the elastic properties will be observed. It can be seen in Figure 5b that an increase of $\sigma^{\prime }/\sigma$ is accompanied by a strong increase of the in-plane (m=[010]) PR ; however only a minor decrease of the out-of-plane (m=[001]) PR is observed when stress is applied in n=[100] (parallel to the inclusion plane). It should be also noted that under these loading conditions the Poisson’s ratio value is positive for all $\sigma^{\prime }/\sigma$ values. Thus, in this case the effects in Yukawa system are significantly larger. However, one should stress that this is not a general rule, what is reflected in the study of the hard system with hard nanochannel inclusions [97].

In conclusion, one can see that the differences in elastic properties between both very simple (HS and Yukawa) systems under the same external pressure ($p^{*}=100$) show that effects of geometry are neither the only nor the dominating ones with regard to their influence on Poisson’s ratio of these models.

## 5. Conclusions

It is known that structural modifications at atomic level impact elastic properties of simple models [89,90]. In this work an influence of inclusions in the form of a stack of nanolayers on elastic properties has been studied by MC simulations for a model nanocomposite system with purely geometrical interactions. The crystalline phase of HSs of diameter σ constituted the matrix of that nanocomposite. The nanolayers oriented perpendicularly to [001]-direction were formed by identical HSs of diameter $\sigma^{\prime }$. When $\sigma^{\prime }=\sigma$ one gets a cubic f.c.c. phase. It has been shown that for $\sigma^{\prime }/\sigma \neq 1$ the system changes its symmetry from cubic to tetragonal one, because of that change the values of Poisson’s ratio (average, as well as in particular directions) typically increase. However, the directions (e.g., [100][001]) in which the increase of $\sigma^{\prime }/\sigma$ causes a decrease of the Poisson’s ratio have also been found. The analysis of the Poisson’s ratio surfaces plotted in spherical coordinates revealed that partially auxetic properties are present for all $\sigma^{\prime }/\sigma$ values. Further analysis of the Poisson’s ratio in some particular directions showed that introduction of the nanolayer inclusions causes an increase of the negative Poisson’s ratio in the $\left[101\right]\left[\bar{1}01\right]$-directions for the $\sigma^{\prime }/\sigma$ values in the range between 1 and 1.03. A decrease of the Poisson’s ratio in these directions is observed for $\sigma^{\prime }/\sigma >1.03$. This behavior is qualitatively different from the one observed e.g., in $\left[110\right]\left[1\bar{1}0\right]$-directions, which are equivalent to the former ones in the cubic system. Namely the Poisson’s ratio in the $\left[110\right]\left[1\bar{1}0\right]$-directions reaches zero value (within the experimental error) for $\sigma^{\prime }/\sigma ∼1.02$. The minimal Poisson’s ratio, equal to ν=−0.11, was observed in the tetragonal systems at $\sigma^{\prime }/\sigma =1.054$. This minimum value was almost two times smaller compared to the cubic system without the nanolayers (ν=−0.059) at the pressure $p^{*}=100$. Studies at lower pressures (from $p^{*}=50$ to 11.67) showed that this effect weakens along with the decrease of pressure and becomes hard to be noticed near melting. We relate this observation to the fact that at low pressures, distances between the positions of particles are large enough to compensate size changes of the inclusion particles.

The difference in the elastic properties of Yukawa and HS crystals, both containing periodic inclusions of HS nanolayers, and studied under the same pressure conditions $p^{*}=100$, shows that the effect of auxeticity enhancement appears to be significantly weaker for hard than for Yukawa interactions. However, one should be aware, this is not a general rule. When considering inclusions in the form of periodic array of [001]-nanochannels in HS systems, a substantially higher increase of partial auxeticity in comparison to analogous Yukawa system was observed [97]. Thus, an important conclusion can be made that geometrical effects are neither the only nor the dominating ones influencing Poisson’s ratio of the studied models. Apart from the interaction potential, the form (shape) of the inclusions and their distribution in the crystalline lattice are the key factors that also exert changes in elastic properties of these models. Thus, it is important to study other models with different types of interactions and different shapes of inclusions. Such research has been conducted and will be presented elsewhere.

The results presented in this manuscript show that relatively small changes of sizes of selected particles forming nanoinclusions can constitute an efficient method to modify the elastic properties of nanocomposites at high pressures. Even though only hard interactions were considered in the studied system, the obtained results may be useful to researchers in the field of materials engineering and smart materials. The authors hope that some of the results presented here will constitute a source of inspiration for real experiments.

## Abbreviations

The following abbreviations are used in this manuscript (listed in order of occurrence in the text):

f.c.c.	face-centered cubic lattice
PR	Poisson’s ratio
HS	Hard Sphere
MC	Monte Carlo
